# Identification of novel biomarkers involved in doxorubicin-induced acute and chronic cardiotoxicity, respectively, by integrated bioinformatics

**DOI:** 10.3389/fcvm.2022.996809

**Published:** 2023-01-11

**Authors:** Hongyan Qian, Yi Qian, Yi Liu, Jiaxin Cao, Yuhang Wang, Aihua Yang, Wenjing Zhao, Yingnan Lu, Huanxin Liu, Weizhong Zhu

**Affiliations:** ^1^Department of Pharmacology, School of Medicine and School of Pharmacy Nantong University, Nantong, China; ^2^Cancer Research Center Nantong, Nantong Tumor Hospital and Tumor Hospital Affiliated to Nantong University, Nantong, China; ^3^School of Overseas Education, Changzhou University, Changzhou, China; ^4^Shanghai Labway Medical Laboratory, Shanghai, China

**Keywords:** doxorubicin, cardiotoxicity, RNA-seq, biomarkers, mice

## Abstract

**Background:**

The mechanisms of doxorubicin (DOX) cardiotoxicity were complex and controversial, with various contradictions between experimental and clinical data. Understanding the differences in the molecular mechanism between DOX-induced acute and chronic cardiotoxicity may be an ideal entry point to solve this dilemma.

**Methods:**

Mice were injected intraperitoneally with DOX [(20 mg/kg, once) or (5 mg/kg/week, three times)] to construct acute and chronic cardiotoxicity models, respectively. Survival record and ultrasound monitored the cardiac function. The corresponding left ventricular (LV) myocardium tissues were analyzed by RNA-seq to identify differentially expressed genes (DEGs). Gene Ontology (GO), Kyoto Encyclopedia of Gene and Genome (KEGG), and Gene Set Enrichment Analysis (GSEA) found the key biological processes and signaling pathways. DOX cardiotoxicity datasets from the Gene expression omnibus (GEO) database were combined with RNA-seq to identify the common genes. Cytoscape analyzed the hub genes, which were validated by quantitative real-time PCR. ImmuCo and ImmGen databases analyzed the correlations between hub genes and immunity-relative markers in immune cells. Cibersort analyzed the immune infiltration and correlations between the hub genes and the immune cells. Logistic regression, receiver operator characteristic curve, and artificial neural network analysis evaluated the diagnosis ability of hub genes for clinical data in the GEO dataset.

**Results:**

The survival curves and ultrasound monitoring demonstrated that cardiotoxicity models were constructed successfully. In the acute model, 788 DEGs were enriched in the activated metabolism and the suppressed immunity-associated signaling pathways. Three hub genes (Alas1, Atp5g1, and Ptgds) were upregulated and were negatively correlated with a colony of immune-activating cells. However, in the chronic model, 281 DEGs showed that G protein-coupled receptor (GPCR)-related signaling pathways were the critical events. Three hub genes (Hsph1, Abcb1a, and Vegfa) were increased in the chronic model. Furthermore, Hsph1 combined with Vegfa was positively correlated with dilated cardiomyopathy (DCM)-induced heart failure (HF) and had high accuracy in the diagnosis of DCM-induced HF (AUC = 0.898, *P* = 0.000).

**Conclusion:**

Alas1, Atp5g1, and Ptgds were ideal biomarkers in DOX acute cardiotoxicity. However, Hsph1 and Vegfa were potential biomarkers in the myocardium in the chronic model. Our research, first, provided bioinformatics and clinical evidence for the discovery of the differences in mechanism and potential biomarkers of DOX-induced acute and chronic cardiotoxicity to find a therapeutic strategy precisely.

## Introduction

Cardiovascular disease and cancer rank as the two leading premature causes of death worldwide (1). In the last decade, the survival of patients with cancer has greatly improved due to the improvement of comprehensive treatment, especially the tremendous development and application of anticancer drugs (2). However, the following problem is that due to prolonged survival, complications caused by various cancer therapies appear, especially the toxicity of anticancer drugs to the heart (3). In fact, there are very few interventions available to address the problem. Therefore, it is particularly important to discover the mechanisms involved in the cardiotoxicity induced by these anticancer drugs and to find potential biomarkers.

Doxorubicin (DOX), as the first-line chemotherapy drug for various cancers, has limited availability since its cardiotoxicity was first reported in 1979 (4). Its cardiotoxicity is divided into three categories based on the time of onset. The first is acute cardiotoxicity, which occurs within 2 weeks after a single chemotherapy regimen. The second is early-onset chronic cardiotoxicity, which occurs within 1 year after stopping the treatment, usually manifesting as heart failure (HF) caused by dilated cardiomyopathy (DCM). The third is late-stage chronic cardiotoxicity, which develops years or even decades after the end of chemotherapy (5). The incidence rate of acute cardiotoxicity and chronic one is 11–21 and 1.7%, respectively (6, 7). Their pathophysiological changes are complex, with both similarities and differences. Though the underlying mechanisms such as oxidative stress, lipid peroxidation, topoisomerase II inhibition, DNA binding and alkylation, dysregulation of the cardiomyocyte-specific genes, inflammatory cytokines, necroptosis, autophagy, direct membrane damage, dysfunction of adrenergic receptors, and misregulation of calcium handling have been found for DOX-induced cardiotoxicity (8–10), the prevailing paradigm holds that oxidative stress is the key mechanism that is associated with mitochondrial dysfunction and cardiomyocyte death in both models (11). Acute cardiotoxicity includes myocardial rupture, cardiomyocyte atrophy, and vacuolar pro-apoptotic cells. The chronic model can lead to left ventricular (LV) dysfunction and typical DCM, which can affect the ventricles and atria, dilate the heart muscles and chamber, and eventually lead to HF (7, 12, 13). In fact, the potential differences are far more than these, and there is a lack of biomarkers for their respective classifications. Therefore, comprehensively understanding these differences and finding the appropriate biomarkers will have a profound impact on clinical management and prognosis.

The rapid development of microarray and sequencing technologies has revolutionized the depth of research and the complexity of collecting and examining molecular data in current biomedical research (14). Gene expression omnibus (GEO) database provides flexible mining tools that enable users to easily query and download data in the context of their specific interests (15, 16). For example, by using the GEO database *via* GEO2R, Qin et al. (17) have revealed the potential roles of HMOX1 in DOX-induced cardiotoxicity. However, the study has only focused on acute cardiotoxicity.

Herein, we established the acute and chronic mouse models, respectively, and used RNA-seq data as the training group to identify differentially expressed genes (DEGs) for further analysis. The molecular mechanisms were addressed by the Gene Ontology (GO) function, Kyoto Encyclopedia of Gene and Genome (KEGG) pathways, Gene Set Enrichment Analysis (GSEA), protein–protein interaction (PPI) network, and Cytoscape analysis. RNA expression datasets for DOX-induced cardiotoxicity were downloaded from the GEO databases as the testing group to acquire common DEGs with our RNA-seq. We further identified the hub genes and validated the experiments. Finally, we analyzed the correlations between hub genes and immune cells. The clinical database from the GEO database as the validation group verified the predictive effect of the hub genes. Our findings provided further insights into the mechanisms underlying the progression of DOX-induced acute and chronic cardiotoxicity and suggested that hub genes are potential diagnostic biomarkers in the myocardium.

## Materials and methods

### Mouse models of DOX-induced acute and chronic cardiotoxicity

Animal studies were approved by the Institutional Animal Care and Use Committee of Nantong University. In the acute model, 6–8-week-old male C57BL/6 mice were injected intraperitoneally with 20 mg/kg of DOX once (*n* = 23) or saline solution (*n* = 10), based on a previous study (18). Mice were euthanized on day 4 after the first injection, and the LV tissues were stored in liquid nitrogen. In the chronic model, mice (*n* = 15) were injected intraperitoneally with 5 mg/kg of DOX weekly for 3 continuous weeks, with reference to a previous report (19). Then, the mice were euthanized and the LV tissues were stored in liquid nitrogen for 6 weeks after the first injection. The same protocols for each model were reproductive for survival analysis.

### Cardiac function evaluation by echocardiography

The LV function was evaluated with transthoracic echocardiography before the mice were euthanized. Blind tests were performed on the treatment and control groups using echocardiography. With a high-resolution ultrasound frequency-imaging platform (Vevo 2100 System), echocardiography was performed 10 min after initiation of sedation to limit anesthesia-induced impairment of cardiac function. The percentages of LV ejection fraction (EF) and fractional shortening (FS) were calculated from the echocardiography data, as previously described (20).

### RNA sequencing data

Nine LV tissues of each group (NC group, acute DOX group, and chronic DOX group) were divided into triplicates randomly. Total RNA was extracted by Trizol reagent (Invitrogen, USA), and the purity was evaluated with an Agilent 2100 Bioanalyzer. Subsequently, the library constructions were made using 1 μg of total RNA with RIN > 6.5. Next-generation sequencing library preparations were constructed according to the manufacturer’s protocol. The prepared libraries were then subsequently multiplexed and loaded on an Illumina HiSeq instrument. Sequencing was carried out using a paired-end configuration. Image analysis and base calling were conducted by the HiSeq instrument. To remove the technical element, Cutadpt (V1.9.1) was used to process pass-filter data in FASTQ format, converting it into high-quality, clean data. Differential expression analysis employed the DESeq2 Bioconductor package, and regularized logarithm was used as the standardized method. The | fold change| (| FC|) > 2 and Benjamini-Hochberg-adjusted *p*-value of genes were set at < 0.05 to detect DEGs.

### GEO data download

The keywords ‘‘anthracycline cardiotoxicity,’’ ‘‘DOX cardiotoxicity,’’ or ‘‘cardiotoxicity’’ were used to search for the GEO Datasets. The ‘‘GEO query’’ package in R software was used to download expression profiling by microarray datasets of DOX-induced cardiotoxicity (GSE59672, GSE23598, GSE2965, and GSE120895) from the GEO database. The microarray datasets GSE59672^1^ and GSE23598^2^ presented the acute model, whose DEGs were screened by | FC| > 1.5 and *P* < 0.05. In GSE59672 (3 model samples vs. 3 control samples), the whole hearts of mice were selected for RNA extraction and hybridization on Affymetrix microarrays at day 5 after a single intraperitoneal injection of 15 mg/kg DOX (or saline solution). In GSE23598 (2 model samples vs. 2 control samples), the model construction method was the same as GSE59672, except that the heart tissues were collected on day 4. The GSE2965^3^ showed chronic models (2 model samples vs. 2 control samples), where mice were injected with 3 mg/kg DOX weekly for 12 weeks and their hearts were harvested at 12 and 18 weeks after the first injection. The DEGs of GSE2965 were screened by | FC| > 1.5. The GSE120895^4^ showed human endomyocardial biopsies (47 DCM patients showing HF symptoms vs. eight individuals with normal LVEF), which was profiled to identify possible biomarkers sensitive to HF.

### Establishment of DOX-treated primary cardiomyocytes of the adult rat model

Rat cardiomyocytes were isolated as previously described (21). Adult rat cardiomyocytes were isolated by Langendorff perfusion and Type II collagenase digestion and cultured in the serum-free Medium199 for 4 h before DOX treatment. After culturing these cardiomyocytes in Medium199 with 2 μM DOX for 24 h, the cells were harvested for further analysis.

### Quantitative real-time PCR (qPCR)

RNA was isolated from heart tissues or cardiomyocytes by using Trizol. Reverse transcription and quantitative PCR were carried out by using a two-step PrimeScriptTM RT reagent kit (TAKARA), and a QuantStudio3 Real-Time PCR System (Applied Biosystems, Thermo Fisher Scientific, United States) was used for qPCR. Primers for the genes were synthesized and obtained from Thermo Fisher Scientific. The primer sequences are presented in Supplementary Table 1.

### Bioinformatics analysis

The Limma software package in R^5^ was employed to screen DEGs of GEO data. The GO and KEGG analyses were performed using the cluster profiler package in R (22). A *P-*value of < 0.05 was set as the cut-off criterion for significance. The GSEA was used to associate genes with possible pathways. A false discovery rate (FDR) of < 0.5 and *P* < 0.05 were used as the criteria for judging statistical significance. Interactive relationships and PPI networks of the DEGs were evaluated using the STRING database. The Cytoscape software 3.2.2 was used to construct and visualize a biological network of key DEGs. The Clustering Coefficient and DMNT method in the CytoHubba were used to find the hub genes. Tabula Muris database^6^ analyzed the location of hub genes in the normal mouse heart based on single-cell transcriptome data. ImmuCo database^7^ and ImmGen database^8^ analyzed the gene co-expression and correlation in immune cells. Cibersort analyzed immune infiltration.

### A receiver operating characteristic curve analysis

The receiver operating characteristic curve was used to analyze diagnostic values of hub genes, including the area under the curve (AUC) and significance obtained from the GSE120895 dataset.

### Artificial neural network analysis

Artificial neural networks are complex computational models that can implement machine learning and pattern recognition. The key pretreatment variables identified as having diagnostic significance by ROC and logistic regression were chosen as input variables for our ANN. These variables were VEGFA, HSPH1, and VEGFA combined with HSPH1. The multilayer perceptron analysis (MLP) used the normalized and log2 transformed gene expression data. The complete procedure for MLP analysis was performed with reference to a previous report (23). All models were trained with a randomly selected subset of 70% of the patients, while the other 30% was used to test each model. A minimum number of one up to a maximum of 50 iterations was established for each network. There was only one hidden layer with two units using hyperbolic tangent activation functions. The output layer used softmax activation functions. This analysis was performed in IBM SPSS Statistics Version 17.

### Statistical analysis

Limma package, ClusterProfiler package, and Cytoscape V3.8.2 software were used to analyze RNA-seq or GEO data. SPSS V17 and GraphPad V8.0.2 software were used for the statistical analysis of experimental or clinical data. Unpaired Student’s *t*-test or one-way ANOVA test was used to compare the difference between two or three groups. Correlations were determined by Pearson analysis. The logistic regression analyzed the relationship between the hub genes and DCM. The difference was considered statistically significant if *P < 0.05, ^**^P < 0.01, and ^***^P < 0.001.

## Results

### Assessment of DOX-induced cardiotoxicity models

**FIGURE 1:**
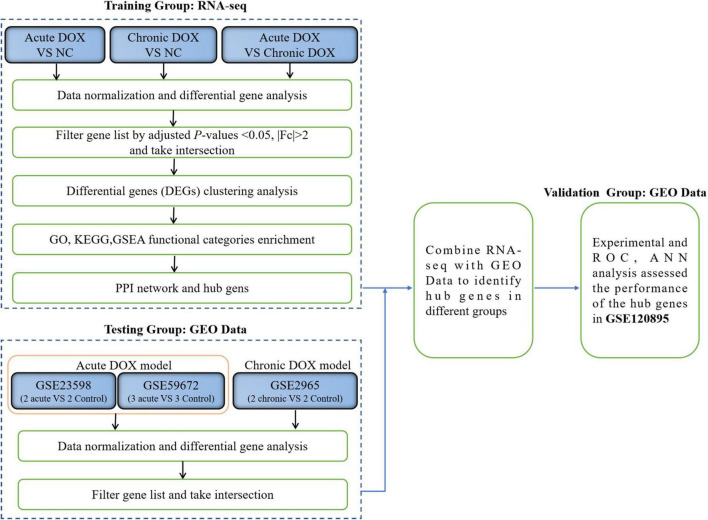
Flow chart of the study.

**FIGURE 2:**
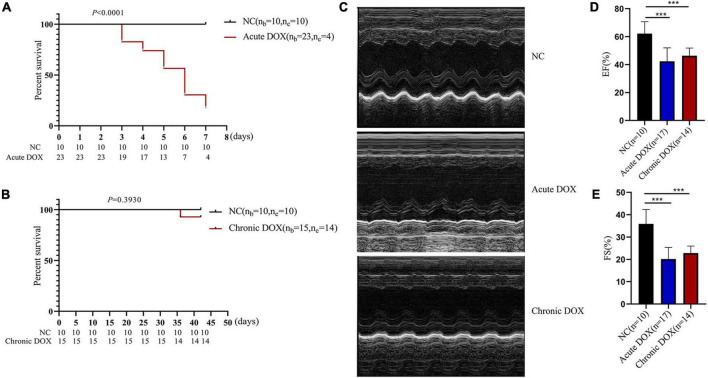
Detecting DOX-induced acute and chronic cardiotoxicity of mouse model. **(A)** Survival analysis of the acute model. **(B)** Survival analysis of the chronic model. **(C)** Representative echocardiography images of the normal control (NC), DOX-induced acute cardiotoxicity group (acute DOX), DOX-induced chronic cardiotoxicity group (chronic DOX). **(D,E)** The percentages of EF and FS were calculated from the echocardiography data. ***P < 0.001, as calculated by one-way ANOVA.

The workflow of this research was shown in Figure 1. To explore the different mechanisms of DOX-induced acute and chronic cardiotoxicity, mouse models were constructed and assessed. The Kaplan–Meier survival analysis showed that mice began to die 3 days after injection, and four mice (4/23) barely survived on day 7 in the acute model (vs. normal control, *P* < 0.0001), whereas only one mouse (1/15) died after 35 days post the first injection in the chronic model (Figures 2A,B). However, echocardiographic data displayed that the EF and FS of the living mice before sacrifice in the acute model sharply declined by about 20% (42.36 vs. 62.14% in the normal control) and by about 15% (20.18 vs. 35.91% in the normal control), respectively. While EF and FS of the mice before sacrifice in the chronic model decreased by about 15% (46.35 vs. 62.14% in the normal control) and 13% (22.85 vs. 35.91% in the normal control), respectively (Figures 2C–E). These results illustrated that cardiotoxicity was presented in both models, evidenced by EF < 50% or a 10% decrease from baseline. However, the acute model mice appeared to have much more severe cardiotoxicity in a shorter time and higher mortality than the chronic one.

### Identification of the key biological processes and signaling pathways in DOX-induced cardiotoxicity

**FIGURE 3:**
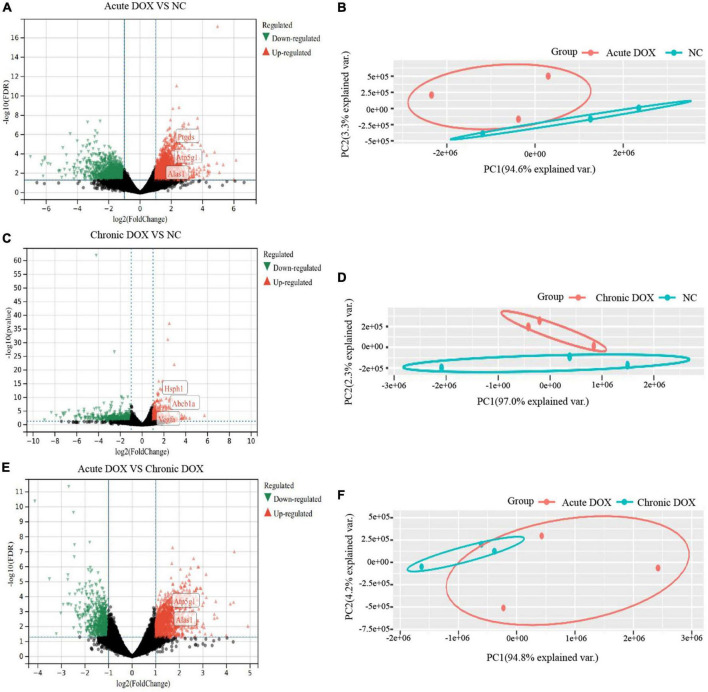
Identification of differently expressed genes in RNA-seq. **(A,C,E)** Volcano plots of differently expressed genes (DEGs) in RNA-seq. Green: down-regulated genes; Gray: no DEGs; Red: up-regulated genes. **(B,D,F)** Principle component analysis (PCA). Total RNA was extracted and subjected to RNA sequencing analysis. PCA shows distinct patterns among groups. *n* = 3 of each group.

**FIGURE 4:**
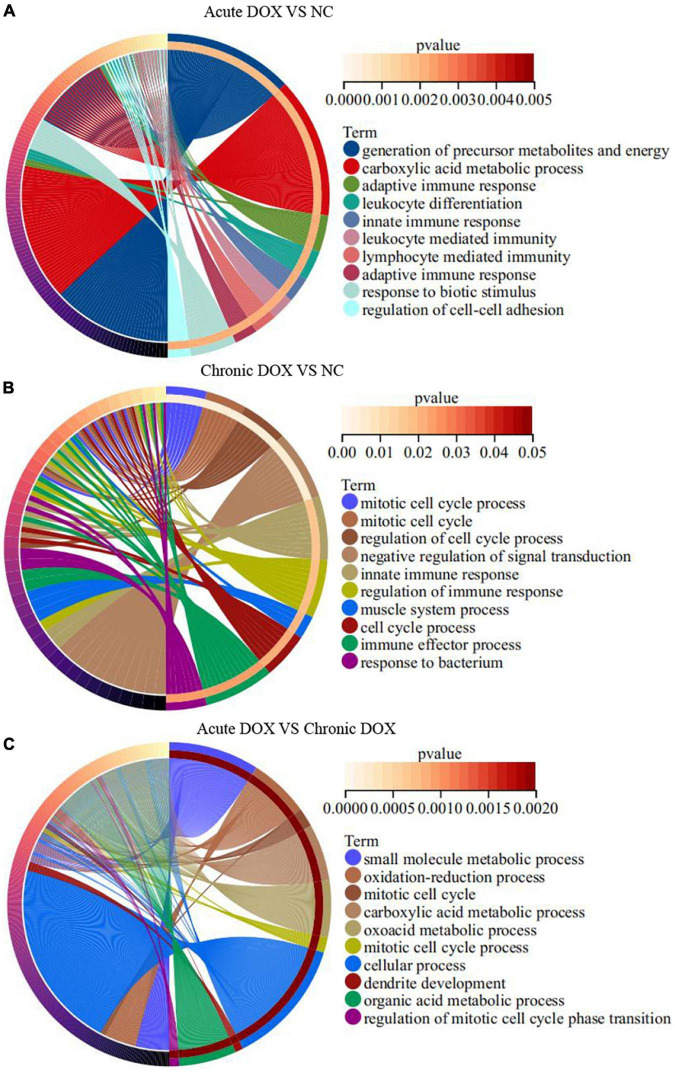
GO enrichment analyses of DEGs in TOP10 biological process. **(A)** Acute DOX vs. NC. **(B)** Chronic DOX vs. NC. **(C)** Acute DOX vs. Chronic DOX.

RNA-seq was carried out to understand the distinct mechanisms behind the two models. In comparison with normal control, 788 DEGs (212 down, 576 up) in the acute group and 281 DEGs (113 down, 168 up) in the chronic group were found. A total of 713 DEGs (220 down, 493 up) were displayed by comparing the acute group with the chronic group (Figures 3A,C,E). Then, principal component analysis (PCA) demonstrated that samples from the three groups were significantly separated from each other and clustered well (Figures 3B,D,F). Furthermore, GO analysis for BP in the acute model showed TOP10 terms (297 DEGs) that focused on the generation of precursor metabolites and energy, carboxylic acid metabolic process, adaptive immune response, leukocyte differentiation, innate immune response, etc. Noticeably, metabolism-associated DEGs were about 50% (146/297) and immune-regulation-associated DEGs were about 30% (87/297) (Figure 4A and Supplementary Table 2). The GO analysis for the chronic model showed that the TOP10 terms (135 DEGs) focused on the cell-cycle process and negative regulation of signal transduction, which was about 40% (48/135) and 13% (18/135), respectively (Figure 4B and Supplementary Table 2). Furthermore, metabolic processes, such as the oxoacid metabolic process, carboxylic acid metabolic process, and oxidation–reduction process, were more highlighted in the acute model than in the chronic model (Figure 4C).

**FIGURE 5:**
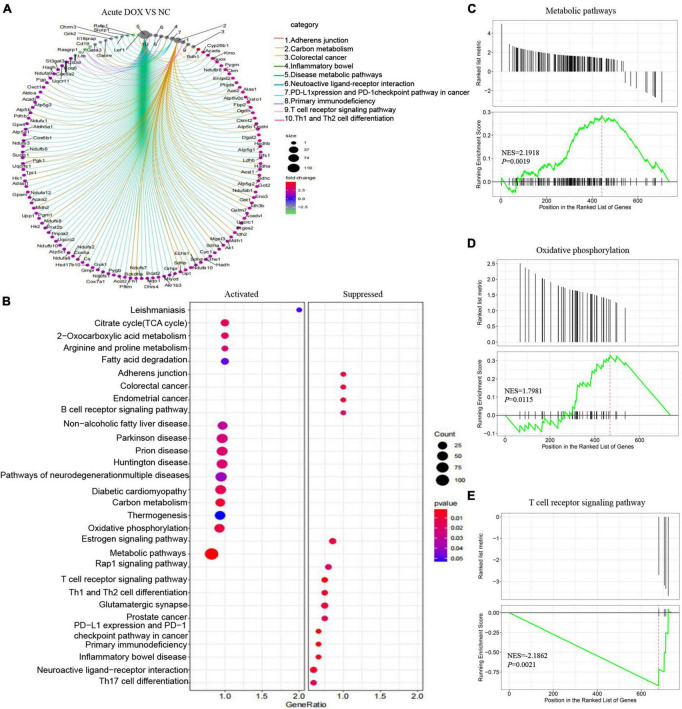
Kyoto Encyclopedia of Gene and Genome and Gene Set Enrichment analyses of DEGs in the acute model. **(A)** The circle graph shows the DEGs enriched in the TOP10 KEGG signaling pathways. **(B)** KEGG analysis showed the activated and suppressed signaling pathways. **(C–E)** GSEA analysis showed various signaling pathways associated with DOX-induced acute cardiotoxicity.

**FIGURE 6:**
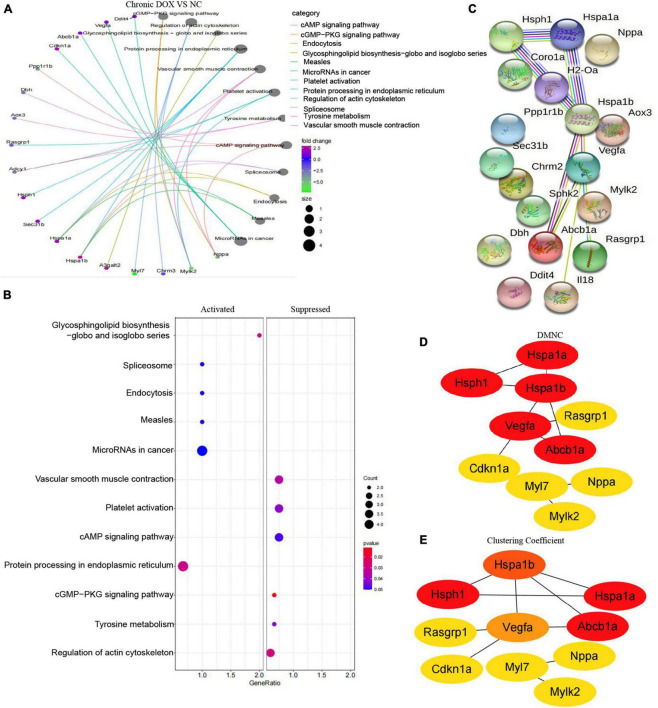
Kyoto Encyclopedia of Gene and Genome and protein–protein interaction network analyses of DEGs in chronic model. **(A)** The circle graph shows the DEGs enriched in the TOP10 KEGG signaling pathways. **(B)** KEGG analysis showed the activated and suppressed signaling pathways. **(C)** STRING database analysis of the DEGs in chronic model. **(D,E)** TOP10 hub genes were screened from the PPI network using the Clustering Coefficient and DMNC methods of the CytoHubba.

In fact, KEGG further validated our findings. KEGG analysis exhibited that metabolism and immunity-associated signaling pathways were the most relevant pathways in the acute model (Figure 5A and Table 1). However, G protein-coupled receptor (GPCR) related signals, such as cAMP and cGMP-PKG signaling pathways, platelet activation, and vascular smooth muscle contraction were the hub signaling pathways in the chronic model (Figure 6A and Table 1). Further analysis revealed that metabolic pathways, such as 2-oxocarboxylic acid metabolism and carbon metabolism, were activated, while immunity-associated signaling pathways, such as the B-cell receptor signaling pathway and T-cell receptor signaling pathway, were suppressed in the acute model (Figure 5B). However, GPCR-related signaling pathways and vascular smooth muscle contraction were suppressed in the chronic model (Figure 6B). In addition to these, GSEA also validated metabolic pathways (NES = 2.1918, *P* = 0.0019) and oxidative phosphorylation (NES = 1.7981, *P* = 0.0115) that were upregulated, while T cell receptor signaling pathway (NES = −2.1862, *P* = 0.0021) was inhibited in the acute model (Figures 5C–E). These results collectively reminded us that activated metabolism and suppressed immunity regulation were the key signaling pathways in acute cardiotoxicity, while GPCR-related signaling pathways were the critical events in the chronic model.

**TABLE 1:** TOP10 KEGG terms of the DEGs in the acute and chronic model, respectively.

ID	Description	Enrichment score	NES	*P-value*	Group
mmu01100	Metabolic pathways	0.284	2.1918	0.0019	Acute
mmu04660	T cell receptor signaling pathway	-0.925	-2.1862	0.0021	Acute
mmu05235	PD-L1 expression and PD-1 checkpoint pathway in cancer	-0.9578	-1.9732	0.0021	Acute
mmu05340	Primary immunodeficiency	-0.9578	-1.9732	0.0021	Acute
mmu05321	Inflammatory bowel disease	-0.8978	-1.8497	0.0042	Acute
mmu04080	Neuroactive ligand-receptor interaction	-0.5771	-2.0047	0.0064	Acute
mmu01200	Carbon metabolism	0.398	2.0649	0.0074	Acute
mmu04658	Th1 and Th2 cell differentiation	-0.779	-1.8412	0.0083	Acute
mmu04520	Adherens junction	-0.9483	-1.6527	0.0101	Acute
mmu05210	Colorectal cancer	-0.9483	-1.6527	0.0101	Acute
mmu04022	cGMP-PKG signaling pathway	-0.8626	-1.5821	0.0118	Chronic
mmu04810	Regulation of actin cytoskeleton	-0.7195	-1.558	0.0289	Chronic
mmu00603	Glycosphingolipid biosynthesis—globo and isoglobo series	0.9811	1.327	0.0292	Chronic
mmu04141	Protein processing in endoplasmic reticulum	0.6189	1.7133	0.0304	Chronic
mmu04270	Vascular smooth muscle contraction	-0.7594	-1.5185	0.0358	Chronic
mmu04611	Platelet activation	-0.751	-1.5015	0.042	Chronic
mmu00350	Tyrosine metabolism	-0.7901	-1.4491	0.0438	Chronic
mmu04024	cAMP signaling pathway	-0.7447	-1.4889	0.0482	Chronic
mmu03040	Spliceosome	0.8555	1.4909	0.0498	Chronic
mmu04144	Endocytosis	0.8555	1.4909	0.0498	Chronic

### Mining the hub genes in DOX-induced acute and chronic cardiotoxicity

**FIGURE 7:**
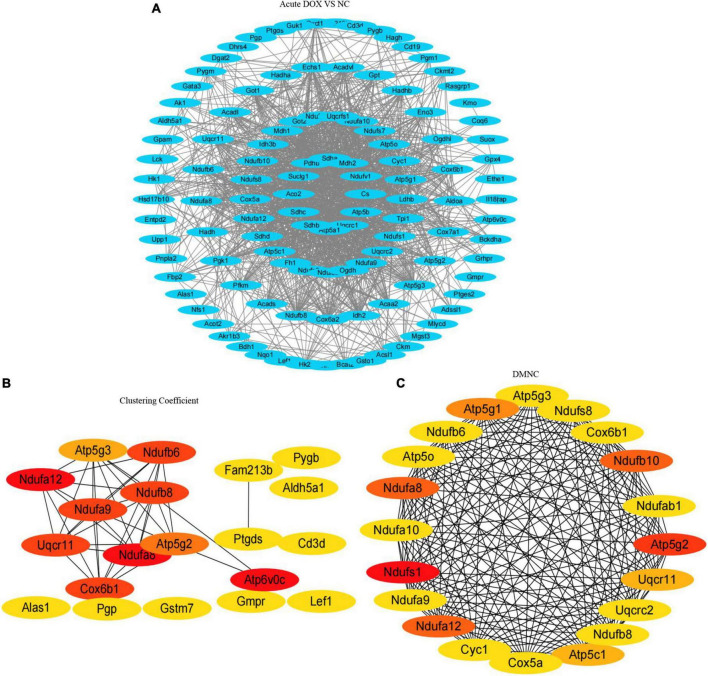
Protein–protein interaction network and hub clustering modules. **(A)** STRING database analysis of the DEGs in acute model. **(B,C)** TOP20 hub genes were screened from the PPI network using the Clustering Coefficient and DMNC methods of the CytoHubba.

As the signaling pathways referred to lots of DEGs, we got the DEGs from the TOP10 signaling pathways of KEGG (Table 1) and systematically analyzed the relationships between them by STRING database and visualized the data using Cytoscape (Figures 6C, 7A). To identify the hub genes, two methods—clustering coefficient and density of maximum neighborhood component (DMNC), of the CytoHubba package in Cytoscape were used to get TOP20 hub genes in the acute model and TOP10 hub genes in the chronic model (Figures 6D,E, 7B,C).

**FIGURE 8:**
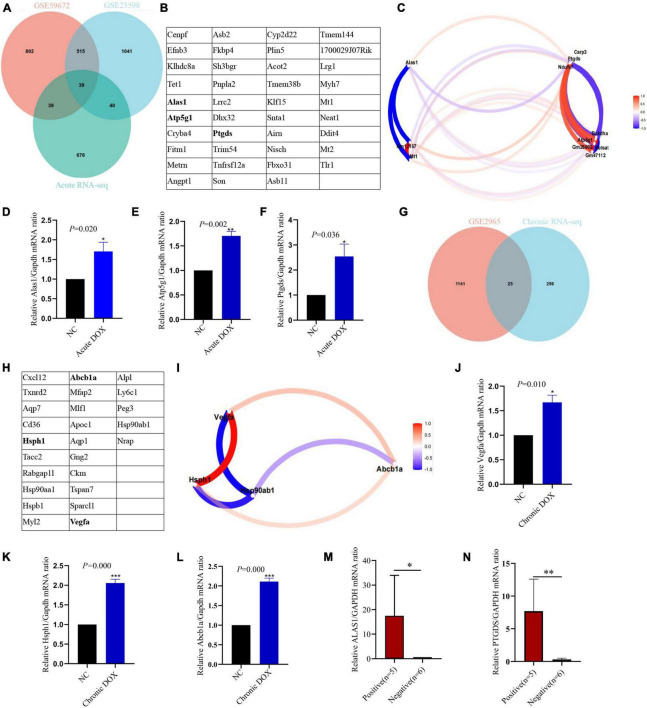
Validation of hub genes in acute and chronic model. An intersection analysis of RNA-seq and GEO databases of acute model **(A)** and chronic model **(G)**. 39 common DEGs of acute model **(B)** and 25 common DEGs of chronic model **(H)** were listed in the table. The genes marked in bold were the hub genes. Gene correlation coefficient diagram showed the relationship between hub genes and DEGs in the acute **(C)** and chronic **(I)** models. qPCR detected the expression of the hub genes in LV tissues of the acute model **(D–F)** and chronic model **(J–L)**. **(M,N)** qPCR detected the expression of the hub genes in the blood (leukocyte) of patients who were undergoing anthracyclines treatment. NC, normal control; positive, anthracyclines -induced cardiac injury patients; negative: normal cardiac function patients; *P < 0.05; ^**^P < 0.01; ^***^P < 0.001; *n* = 3.

To certify our findings, GEO data were analyzed to further validate the above results. As shown in Figures 8A,G, 39 DEGs were identified as common to GSE23589, GSE59672, and RNA-seq data in the acute model, which contained some proven genes, such as Klf15 and Neat1. Meanwhile, 25 DEGs were identified as common to GSE2965 and RNA-seq data in the chronic model, where Ckm had been reported (Figures 8B,H). Furthermore, we analyzed the intersection of the common DEGs and above TOP20 or TOP10 hub genes in our RNA-seq data. Ultimately, we found 3 hub genes (Alas1, Atp5g1, and Ptgds) in the acute model and three hub genes (Hsph1, Abcb1a, and Vegfa) in the chronic model (Figures 8B,H). Furthermore, based on the gene expression matrix, gene correlation coefficient analysis showed that the hub genes had a high correlation in each model (Figures 8C,I).

### Identifying candidate biomarkers in DOX-induced acute cardiotoxicity

To further verify the accuracy of these hub genes, qPCR detected that Alas1, Atp5g1, and Ptgds displayed upregulated tendency in the LV tissues of the acute model, which were consistent with the RNA-seq (Figures 3A, 8D–F). Furthermore, single-cell transcriptome data of the heart from the normal mouse on the Tabula Muris database reminds us that except Abcb1a, the rest of the hub genes is mainly located in cardiac muscle cells (Supplementary Figures 1A–F). Hence, we established the DOX-treated adult rat cardiomyocytes model to examine the findings. qPCR showed that Atp5g1, Alas1, and Ptgds had the same tendency in the cardiomyocytes as one in the tissues (Supplementary Figures 1G–I). In addition to these, we found that Atp5g1, Alas1, and Ptgds also appeared in immune cells, such as leukocyte cells. Now that both GO and KEGG showed that metabolism and immunity were core mechanisms in the acute model, we guessed whether the 3 hub genes were involved in core mechanisms. KEGG database showed that Alas1 is a 5-aminolevulinate synthase located in the mitochondrion and it participated in glycine, serine, and threonine metabolism. Atp5g1 was focused on oxidative phosphorylation, metabolic pathways, ROS, and diabetic cardiomyopathy. Ptgds took part in arachidonic acid metabolism and lipid transport (Supplementary Figures 2–4).

**FIGURE 9:**
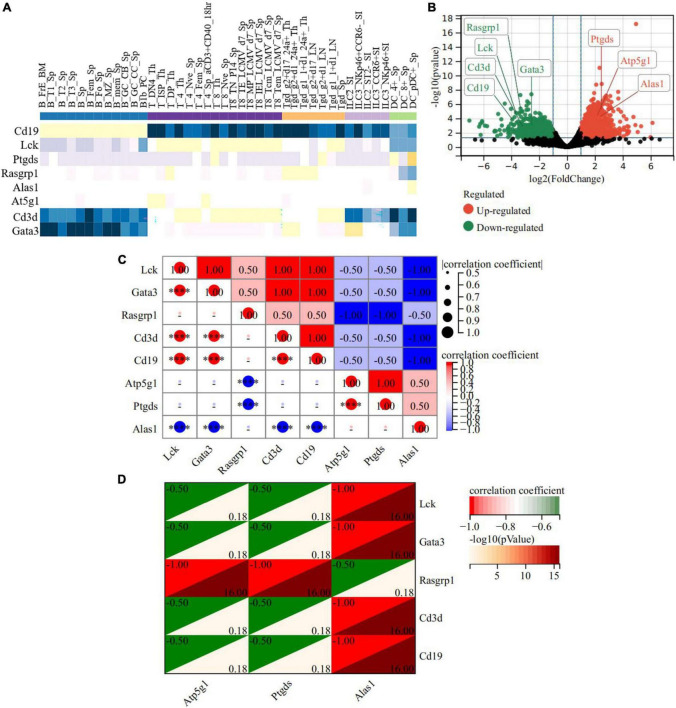
A heat map of the correlation between hub genes and immune-related DEGs in the acute model. **(A)** ImmGen database showed correlation between hub genes and immune-related genes in the immune cell of normal mice. **(B)** The expression of the 3 hub genes and the five immune-related DEGs in RNA-seq of the acute model. **(C,D)** The relationship between Atp5g1 and five immune-related DEGs in the acute model. The horizontal and vertical coordinates represent genes, and different colors represent correlation coefficients. ****P < 0.0001.

We took out the classic immune cell markers involved in our RNA-seq data (Cd3d, Rasgrp1, Lck, Cd19, and Gata3) and analyzed the relationship between the hub genes and these markers. ImmuCo database showed that in normal mouse, the expression of Alas1 was negative with Cd3d in CD8 + T cell (*R* = −0.22, *P* = 0.001), with Cd19 in B cell (*R* = −0.39, *P* = 0.000), and with Gata3 in splenocyte cell (*R* = −0.47, *P* = 0.000). Atp5g1 was negative with Lck in CD8 + T cell (*R* = −0.17, *P* = 0.007), Cd19 in B cell (*R* = −0.44, *P* = 0.000), and Gata3 in splenocyte cell (*R* = −0.40, *P* = 0.000). Ptgds was negative with Gata3 in the splenocyte cell (*R* = −0.03, *P* = 0.720) and Rasgrpl in DC cell (*R* = −0.04, *P* = 0.421) (Supplementary Figure 5). To confirm this finding, the ImmGen database further declared that three hub genes were downregulated while these immune markers had a contrary tendency in the CD8 + T cell, CD4 + T cell, B cell, and DC cell of a normal mouse, reminding us that the three hub genes may negatively regulate immune activation (Figure 9A). Importantly, in the RNA-seq data of the acute model, the three hub genes were upregulated while the five immune markers were downregulated (Figure 9B). Expression matrix correlation analysis also showed that Alas1 was negatively correlated with Gata3, Lck, Cd19, and Cd3d significantly (correlation coefficient = −1, −log10 (*P*-value) = 16), while Atp5g1 and Ptgds were negatively correlated with Rasgrp1 (correlation coefficient = −1, −log10 (*P*-value) = 16) (Figures 9C,D). These results demonstrated that the elevated expression of the three hub genes inhibited the activation of some immunocytes in the acute model.

**FIGURE 10:**
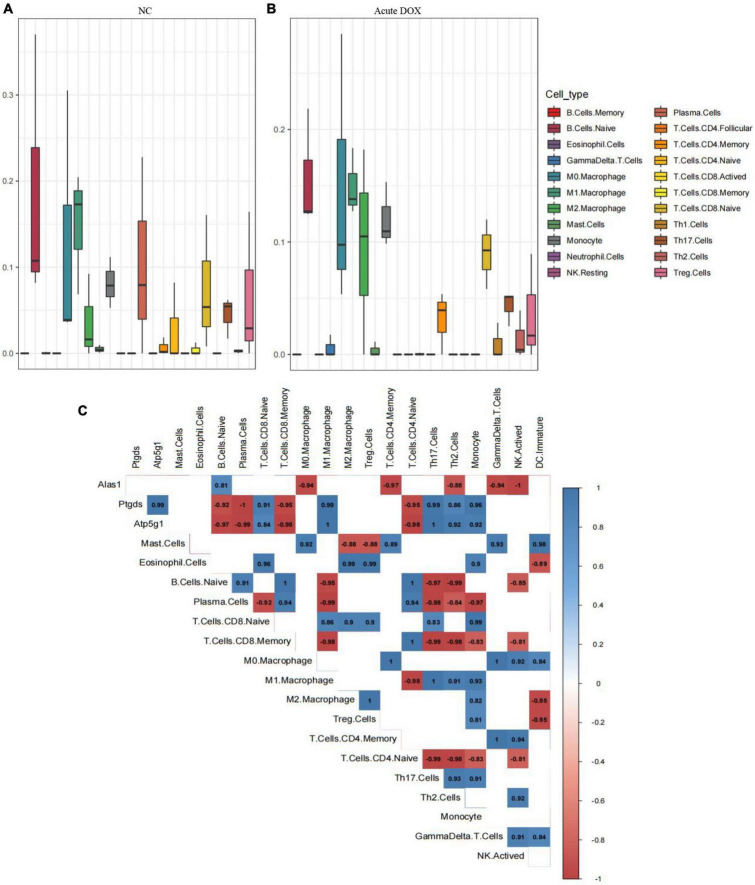
Cibersort analysis for the associations between hub genes and immune cells. **(A)** 22 immune cells infiltration in normal control. **(B)** 22 immune cells infiltration in the acute model. **(C)** The relationship between 3 hub genes with immune cells in the acute model.

Moreover, Cibersort analysis for 22 mouse immune cells illustrated that the proportion of monocyte, Th1 cell, and activated NK cell were slightly improved from 8.1 to 12%, 0 to 0.9%, and 2.9 to 3.9%, respectively. This suggested that inflammation appeared to be involved in acute cardiotoxicity, which was generally consistent with the previous report (24, 25). Notably, M0 macrophages were slightly improved from 12.7 to 14.6% but the M1 macrophage remained unchanged. However, M2 macrophage obviously increased about 2 times—from 3.6 to 9.6%—and the plasma cell sharply decreased from 10.2% to about 0. In addition, the B cells naive, Treg cells, and CD4 memory T Cells were all decreased more or less, revealing immunosuppression appeared in the acute model (Figures 10A,B). These results declared that the immunosuppressive feature was more obvious than inflammation in the acute model. In addition to these, the correlation between the three hub genes with immunity cells showed that Alas1 was negatively correlated with activated NK cell, Gamma delta T cell, CD4 memory T cell, and M0 macrophage. Both Ptgds and Atp5g1 were negative with M1 macrophage, B cells naive, plasma cell, and CD8 memory T cell (Figure 10C). All of the results strongly demonstrated that Alas1, Atp5g1, and Ptgds were candidate biomarkers for DOX-induced acute cardiotoxicity as they dominated metabolism and immunity simultaneously.

### Testing potential biomarkers for DOX-induced chronic cardiotoxicity

**FIGURE 11:**
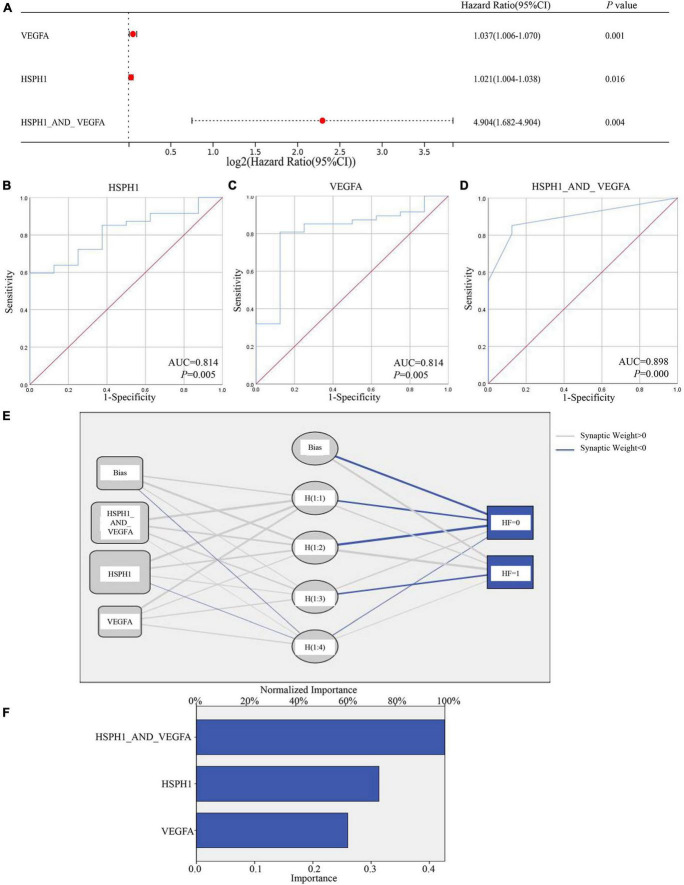
Testing the hub genes of the chronic models by GSE120895. **(A)** Logist regression analysis of factors associated with DCM-induced HF. **(B–D)** ROC curve analysis showed HSPH1 and VEGFA as biomarkers that predicted DCM-induced HF. **(E,F)** ANN analysis of the important role of HSPH1 and VEGFA in predicting DCM-induced HF.

Analogously, qPCR showed the hub genes Hsph1, Vegfa, and Abcb1a were increased in the LV tissues of the chronic model, whose trends were consistent with RNA-seq data (Figures 3C, 8J–L). While qPCR detected that only Hsph1 was elevated significantly in the DOX-treated cardiomyocytes (Supplementary Figures 1J–L), we considered that though Vegfa mainly came from the cardiac muscle cell, it was a secreted protein that led to the detection of no significant changes in the cell. In addition, Abcb1a mainly came from endothelial cells, so it was understandable that no significant changes were detected in cardiomyocytes with DOX treatment. To explore whether these hub genes were the potential biomarkers in the chronic model, we took patients with DCM-induced HF data (GSE120895) as the validation group. The GSE120895 dataset was chosen for two reasons (1) DCM was a typical pathological alteration in DOX-induced chronic cardiotoxicity (5, 7, 12, 13). Liang et al. (2) There was so little clinical data or limited sample capacity of DOX-induced chronic cardiotoxicity in the GEO database that we had to employ DCM GEO data for validation. Logistic regression analysis reminded us that HSPH1 combined with VEGFA (HR = 4.904, *P* = 0.004) was positively associated with chronic DCM-induced HF (Figure 11A). Based on logistic regression analysis, ROC analysis demonstrated that HSPH1 (AUC = 0.814, *P* = 0.005) and VEGFA (AUC = 0.814, *P* = 0.005) had high accuracy in diagnosing chronic DCM-induced HF, especially HSPH1 combined with VEGFA (AUC = 0.898, *P* = 0.000) (Figures 11B–D). Furthermore, ANN analysis confirmed that HSPH1 combined with VEGFA took a significant proportion in monitoring chronic DCM-induced HF (Figures 11E,F). In fact, we did not find the expression of ABCB1a in this database. It was speculated that ABCB1a is a membrane-associated protein, whose level was too low to be detected.

In addition, we analyzed 713 DEGs by comparing the acute model with the chronic one. KEGG further proved that metabolism was dramatically activated in the acute model. Venn analysis showed overlapping between the TOP20 hub genes from the acute vs. the chronic model and TOP20 hub genes from the acute model. All of the 13 common genes were located in metabolism, including Alas1 and Atp5g1 (Supplementary Figure 6). All these results strongly declared that activation of metabolism was the distinctive feature in DOX-induced acute cardiotoxicity. Furthermore, we tested these five biomarkers in the blood (leukocyte) from 11 patients with cancer by qPCR, who were undergoing anthracyclines treatment and had no underlying cardiovascular disease before this therapy. Among these samples, five patients appeared to have abnormal ECG or myocardial enzymes (positive group), while six patients did not show these clinical manifestations (negative group). Exciting results showed ALAS1 and PTGDS were greatly elevated in the positive group (Figures 8M,N), which was highly consistent with our acute model. Indeed, more cases need to be analyzed for further verification.

## Discussion

Both DOX-induced acute and chronic cardiotoxicity can lead to decreased cardiac function and diverse pathologic changes, while the underlying difference in molecular mechanism is still unclear. Exploring the molecular mechanism difference may be an ideal entry point to find their respective biomarkers.

### Activated metabolism and suppressed immunity regulation were the core events in DOX-induced acute cardiotoxicity

The GO and KEGG analysis for the acute model powerfully indicated that the abnormality of metabolic pathways and immunity regulation were the core events. In line with our results, Tan et al. (26) and Ni et al. (27) highlighted several metabolites as potential biomarkers for DOX-induced acute cardiotoxicity. In fact, metabolic disturbances, such as heme metabolism (28), carbon metabolism (29), and oxidative phosphorylation were closely linked to oxidative stress and ROS, which acted as key factors of HF (30). This was also confirmed by our GSEA analysis. Furthermore, the difference between DOX acute and chronic cardiotoxicity was still focused on metabolism. This reminded us again that in addition to addressing oxidative stress and ROS, the importance of metabolism should not be neglected, especially in DOX acute cardiotoxicity.

The DOX-induced immune abnormalities had significant effects on the progression of cardiovascular damage, particularly in the acute phase (31, 32). In the acute model, a seemingly contradictory result showed the coexistence of inflammation and immunosuppression. In fact, the relationship between them is complex, especially in a severe trauma or stressful situations (33, 34). On the one hand, when the inflammatory response persists, it releases both pro- and anti-inflammatory factors to restore the balance. However, the inappropriate expression of these immunosuppressive molecules can aggravate immune suppression (35). On the other hand, patients with severe trauma have a decline in immune function, which makes it difficult to clear the infectious pathogen. This induces the continuous release of pathogen-related molecular patterns or antigens, which in turn act on immune cell pattern recognition receptors or induce adaptive immune activation, leading to persistent inflammatory responses. Thus, the coexistence of inflammation and immunosuppression was not contradictory but a character of serious illness (36). Indeed, in the acute model, we did find that immunosuppressive relative cells were raised substantially, such as M2 macrophage, and a proportion of immune-activating associated cells were sharply decreased, such as Plasma cells and B cells naive. However, Th1 cells, activated NK, and immature DC were increased slightly, which kept the inflammation activation in one of the levels. All these collectively suggested that suppressed immunity regulation is the principal character in DOX acute cardiotoxicity.

### Alas1, Atp5g1, and Ptgds were potential biomarkers in DOX-induced acute cardiotoxicity

Investigating progression-associated gene expression profiles could enrich our understanding of the mechanisms. qPCR verified that three hub genes (Alas1, Atp5g1, and Ptgds), as metabolism-related enzymes, were upregulated in LV tissues of the acute model and DOX-treated cardiomyocytes. Alas1, as a rate-limiting enzyme for heme biosynthesis in the mitochondrial matrix, was associated with coronary artery disease (37). It has been highlighted that elevated heme causes oxidant damage and ROS, which thicks filament proteins to cardiomyocytes contractile dysfunction, breaks DNA strands, and mediates DNA mutations (38, 39). Alas1 was increased when the H9c2 cardiomyocyte was exposed to DOX (40). Hence, we speculated that DOX-induced elevation of Alas1 promoted heme biosynthesis to induce oxidative stress, which destroyed mitochondria and led to mitochondrial DNA damage (39, 41, 42). Atp5g1, as the ATP synthase membrane subunit c locus 1, regulated the mitochondrial permeability transition pore complex (PTPC) (43), and its mutation aggravated PTPC-mediated hypoxia/reoxygenation damages in cardiomyocytes (44). In addition, Atp5g1 is co-localized with the mitochondrial marker HSP60, which is associated with ROS, increased fibrosis, mitochondrial damage, and autophagosomes during HF (45, 46). DOX stimulated calcium release from the mitochondrial matrix through induction of the PTPC, which led to oxidative stress, contributing to mitochondrial bioenergetic failure and cell death (47). Based on our results and from the literature, we guessed elevated Atp5g1 promoted PTPC-induced oxidative stress mediating mitochondrial damage and cell death. Prostaglandin-D2-synthase (Ptgds) was expressed in the atherosclerotic intima and accumulated in the atherosclerotic plaque of coronary arteries with severe stenosis (48).

Intricate relationships between metabolism and immunity were immanent, especially in the mitochondria (49). The abnormal metabolism of immune cells can mediate immune and inflammatory response disorders, where insufficient ATP production aggravates immunosuppression (50, 51). The immune disorder can in turn induce metabolic disorders (52). In line with these reports, we found three hub genes were negatively correlated with Gata3 (53, 54), Lck (55, 56), Cd19 (57), Cd3d (58, 59), and Rasgrp1 (60, 61), which were known markers of immune cells activation in the heart and were decreased DEGs in our RNA-seq. Furthermore, Alas1 was negatively correlated with activated NK, Gamma delta T cell, etc., and both Ptgds and Atp5g1 were negatively correlated with M1 macrophage, B cells naive, plasma cell, etc., suggesting that the three hub genes suppressed the immune cell activation. Remarkably, these results collectively suggested that Alas1, Atp5g1, and Ptgds might be desirable markers as they broadly influenced metabolism and immunity simultaneously in DOX-induced acute cardiotoxicity.

### GPCRs pathway, platelet activation, and vascular smooth muscle contraction were involved as the core events in DOX-induced chronic cardiotoxicity

In the chronic model, cAMP and cGMP-PKG signaling pathways, platelet activation, and vascular smooth muscle contraction were the hub signaling pathways that were suppressed. We and others have demonstrated that cAMP or cGMP, as the important elements of GPCRs, were widely involved in the regulation of cardiac function: both β_1_AR- and β_2_AR-mediated augmentation in cAMP led to the activation of PKA, resulting in positive inotropic and relaxant effects (62, 63). In line with our results, Can Ciric Zdravkovic et al. (29) found that DOX inhibited the cAMP/PKA/SIRT1 pathway, which could be attenuated by a Meteorin-like protein. In fact, we have discovered that Gs biased β_2_AR agonist can reduce DOX-induced chronic cardiotoxicity (data not shown here). The cGMP-PKG signaling protects various myocardial properties, including cell growth and survival, endothelial permeability, cardiac contractility, and cardiovascular remodeling (64). Additionally, platelet activation and the coagulation cascade raise thrombosis, which is the most feared complication of cardiovascular diseases. However, DOX did not induce platelet activation but resulted in apoptosis, which might contribute to thrombocytopenia (65), and vascular smooth muscle sensitivity decreases and uncouples in HF. Patients with HF have augmented vascular tone, which increases cardiac workload, impairs ventricular output, and promotes further myocardial dysfunction. These results suggested that DOX stimulation persistently resulted in abnormal GPCRs signal, coagulation disorders, and impairing ventricular output, which led to cardiac remodeling. Once cardiac remodeling occurs, even drug withdrawal cannot stop this irreversible damage.

### Vegfa and Hsph1 were potential biomarkers in diagnosing DOX chronic cardiotoxicity

Vegfa and Hsph1 were dug out by hub gene analysis, and qPCR proved that they were increased in LV tissues of the chronic model. Consistent with our results, Vegfa was upregulated in myocardial injury-associated ventricular remodeling (66), and it was reported that VEGFA is a biomarker in risk factors that mediated coronary heart disease (67). However, an inconsistent report showed that miR-526b-3p mediates DOX-induced cardiotoxicity by targeting STAT3 to inactivate VEGFA (68). For the inconsistent result, we carefully traced the original literature and found that the inconsistency was generated mainly for two reasons: the mice were treated as DOX-induced acute model and they studied the mechanism based on the endothelial cells, which focused on angiogenesis. In fact, besides its role in angiogenesis, VEGFA is also involved in many other aspects: in a mouse DCM model, Vegfa mRNA and protein levels were strikingly upregulated, whereas there was no increase in capillary density (69). Also, Vegfa overexpression in the context of cardiac injury enabled ectopic cardio-myogenesis but inhibited regeneration at the site of the injury in zebrafish (70). Since DOX-induced chronic cardiotoxicity involved DCM (12) and ventricular remodeling (71), we speculated that DOX elevated Vegfa, which promoted DCM-induced HF. Heat shock protein family H member 1 (HSPH1), as a molecular chaperone, was associated with lipid droplets and was upregulated in atherosclerosis (72). Analogously, we found both VEGFA and HSPH1 were increased in patients with DCM, which was an important pathologic change in DOX-induced chronic cardiotoxicity, and VEGFA combined with HSPH1 showed high accuracy in diagnosing DCM-induced HF.

This study has some limitations. At present, DOX-induced chronic cardiotoxicity gene expression data in the publicly available datasets are relatively deficient. Hence, we took DCM GEO data to replace DOX chronic cardiotoxicity data, though DCM was the typical pathological characteristic. In the future, more DOX-induced chronic cardiotoxicity gene expression data will be needed for further analysis. We used the human database to validate the finding from the mouse model, so there may be species differences here. We validated the hub genes at the transcription level, and the potential mechanism should be further explored.

## Conclusion

In the present study, it is concluded that (1) activated metabolism and suppressed immunity regulation were the core events in DOX acute cardiotoxicity; (2) Alas1, Atp5g1, and Ptgds were potential biomarkers for DOX acute cardiotoxicity, as they contributed to metabolism and immunity-regulation simultaneously; (3) the inhibition of GPCR signaling pathway, platelet activation, and vascular smooth muscle contraction was the key mechanisms in DOX chronic cardiotoxicity; and (4) Vegfa and Hsph1 were potential biomarkers for DOX chronic cardiotoxicity. The study provided bioinformatics and clinical evidence for the discovery of the mechanism difference and potential biomarkers of DOX-induced acute and chronic cardiotoxicity in the myocardium so as to find a more precise therapeutic strategy.

## Data availability statement

Publicly available datasets were analyzed in this study. This data can be found here: GSE23598 (https://www.ncbi.nlm.nih.gov/geo/query/acc.cgi?acc=GSE23598), GSE59672 (https://www.ncbi.nlm.nih.gov/geo/query/acc.cgi?acc=GSE596 72), GSE2965 (https://www.ncbi.nlm.nih.gov/geo/query/acc.cgi?acc=GSE2965), and GSE120895 (https://www.ncbi.nlm.nih.gov/geo/query/acc.cgi?acc=GSE120895).

## Ethics statement

The studies involving human participants were reviewed and approved by the Research Ethics Board of the Tumor Hospital Affiliated to Nantong University. The patients/participants provided their written informed consent to participate in this study. The animal study was reviewed and approved by Animal Care and Use Committee of the Nantong University.

## Author contributions

WZhu: conception and design, financial support, and final approval of the manuscript. HQ, YQ, YLi, JC, and YW: manuscript writing. HQ, JC, YQ, AY, and WZha: experiment research. HQ, YLu, and HL: statistical analysis. All authors contributed to the article and approved the submitted version.

## Footnotes

1
https://www.ncbi.nlm.nih.gov/geo/query/acc.cgi?acc=GSE59672

2
https://www.ncbi.nlm.nih.gov/geo/query/acc.cgi?acc=GSE23598

3
https://www.ncbi.nlm.nih.gov/geo/query/acc.cgi?acc=GSE2965

4
https://www.ncbi.nlm.nih.gov/geo/query/acc.cgi?acc=GSE120895

5
https://bioconductor.org/packages/release/bioc/html/limma.html

6
https://tabula-muris.ds.czbiohub.org/

7
http://immuco.bjmu.edu.cn/

8
http://rstats.immgen.org/DataPage/
