# Supplementary material for: Identification of novel biomarkers involved in doxorubicin-induced acute and chronic cardiotoxicity, respectively, by integrated bioinformatics
Source: Front Cardiovasc Med. 2023 Jan 11;9:996809. doi: 10.3389/fcvm.2022.996809 (PMC9874088; doi:10.3389/fcvm.2022.996809)
Supplement: Supplementary file 1 [file Data_Sheet_1.docx]

**Supplementary Figure 1.** Sing cell transcriptome data of the heart from the normal mouse. **(A-F)** Tabula Muris database detected the expression and location of the six hub genes in normal mouse heart by sing cell transcriptome data. **(G-L)** Validation of the six hub genes in DOX-treated primary cardiomyocytes of adult rats by qPCR. n=3, NC: normal control, *:*P*<0.05, ns: no significance.

**Supplementary Figure 2.** KEGG analysis for Alas1.

**Supplementary Figure 3.** KEGG analysis for Atp5g1.

**Supplementary Figure 4.** KEGG analysis for Ptgds.

**Supplementary Figure 5.** ImmuCo database analyzed the correlation between hub genes and immune-related genes in immune cell of normal mice. **(A)** The relationship between Alas1andfive immune-related DEGs (Gata3, Lck, Cd3d, Cd19, Rasgrp1)in CD8+T cell, B cell, DC and Splenocyte cell. **(B)** The relationship between Atp5g1 and five immune-related DEGs in CD8+T cell, B cell, DC and Splenocyte cell. **(C)** The relationship between Ptgds and five immune-related DEGs in CD8+T cell, B cell, DC and Splenocyte cell.

**Supplementary Figure 6.** KEGG and PPI network analysis of DEGs in acute model vs chronic model. **(A)** KEGG analysis showed the activated and suppressed signaling pathways. **(B, C)** TOP20 hub genes were screened from the PPI network using the Clustering Coefficient and DMNC methods. **(D)** An intersection analysis of TOP genes from different comparing groups. And the table showed the overlapped 13 genes.

**Supplementary Table 1.** The primer sequences showed as following.

| **Gene name** | **Accession** | **Primer Sequence (5**'**-3**'**)**  **F** | **Primer Sequence (5**'**-3**'**)**  **R** | **Species** |
| --- | --- | --- | --- | --- |
| Ptgds | NM_008963 | GCCTCAATCTCACCTCTACCTT | CCTTGGTGCCTCTGCTGAAT | mouse |
| Alas1 | NM_020559 | CGACTCCACCCTCTTCACCC | CGACTGTTGCGAATCCCTTG | mouse |
| Atp5g1 | NM_001161419 | ATTGGCACAGTGTTTGGTAG | TCACATGGCGAAGAGGAT | mouse |
| Hsph1 | NM_001347534 | AAAATGGTGGCGTGGGAATA | GGATGGGACTGAGATGAC | mouse |
| Abcb1a | NM_011076 | TTCATCGACTCGCTAC | TTCCTGGACAACCTTT | mouse |
| Vegfa | NM_001317041 | CACCCACGACAGAAGG | ATCGGACGGCAGTAGC | mouse |
| Gapdh | NM_001289726 | TGTTTCCTCGTCCCGTAG | CAATCTCCACTTTGCCACT | mouse |
| Ptgds | NM_013015 | GGCTCAGACACCTGCTCTACTCC | CCCAAGAGACCCAAGAGGACCAG | rat |
| Alas1 | NM_024484 | CGTTCGGCTGTGTTGGAGGATAC | TGGCAGGGAGGTGGTGAAGATG | rat |
| Atp5g1 | NM_017311 | CCTGAGTAGACCAGAGGCTCCATC | ACTTGGCTGCTGTGTCAATGTCC | rat |
| Hsph1 | NM_001011901 | AGGAGCGACCGAGAGTGTTGG | CATCCTTGCCTCTGAAGTCTGCTG | rat |
| Abcb1a | NM_133401 | TGAAGAGCGGGCAGACGGTAG | CATTGATGGTCCTGATGTCCTGTCC | rat |
| Vegfa | NM_001317043 | CGGTGTGGTCTTTCGTCCTTCTTAG | AGGGATGGGTTTGTCGTGTTTCTG | rat |

F: forward primer; R: reverse primer

**Supplementary Table 2.** TOP10 GO terms for biological processes in acute and chronic model respectively.

| **ID** | **Description** | **Set Size** | **Enrichment Score** | **NES** | **P value** | **Group** |
| --- | --- | --- | --- | --- | --- | --- |
| GO:0006091 | generation of precursor metabolites and energy | 66 | 0.3055 | 2.0116 | 0.0018 | Acute model |
| GO:0019752 | carboxylic acid metabolic process | 80 | 0.282 | 1.9486 | 0.0019 | Acute model |
| GO:0002250 | adaptive immune response | 21 | -0.7589 | -3.5301 | 0.002 | Acute model |
| GO:0002521 | leukocyte differentiation | 20 | -0.5923 | -2.6963 | 0.002 | Acute model |
| GO:0045087 | innate immune response | 20 | -0.6326 | -2.8799 | 0.002 | Acute model |
| GO:0002443 | leukocyte mediated immunity | 13 | -0.7504 | -2.8684 | 0.002 | Acute model |
| GO:0002449 | lymphocyte mediated immunity | 13 | -0.7504 | -2.8684 | 0.002 | Acute model |
| GO:0002460 | adaptive immune response based on somatic recombination of immune receptors built from immunoglobulin superfamily domains | 13 | -0.7505 | -2.869 | 0.002 | Acute model |
| GO:0009607 | response to biotic stimulus | 32 | -0.4834 | -2.6279 | 0.002 | Acute model |
| GO:0022407 | regulation of cell-cell adhesion | 19 | -0.5399 | -2.4055 | 0.0021 | Acute model |
| GO:1903047 | mitotic cell cycle process | 10 | 0.6095 | 2.008 | 0.0034 | Chronic model |
| GO:0000278 | mitotic cell cycle | 11 | 0.5679 | 1.9233 | 0.0036 | Chronic model |
| GO:0010564 | regulation of cell cycle process | 11 | 0.5899 | 1.9978 | 0.0036 | Chronic model |
| GO:0009968 | negative regulation of signal transduction | 18 | 0.4837 | 2.0097 | 0.0043 | Chronic model |
| GO:0045087 | innate immune response | 14 | -0.5799 | -1.695 | 0.0161 | Chronic model |
| GO:0050776 | regulation of immune response | 13 | -0.5707 | -1.6344 | 0.0176 | Chronic model |
| GO:0003012 | muscle system process | 13 | -0.5701 | -1.6325 | 0.0189 | Chronic model |
| GO:0022402 | cell cycle process | 16 | 0.4662 | 1.8834 | 0.0203 | Chronic model |
| GO:0002252 | immune effector process | 16 | -0.5449 | -1.6347 | 0.0225 | Chronic model |
| GO:0009617 | response to bacterium | 13 | -0.5617 | -1.6086 | 0.023 | Chronic model |
