# Supplementary figures and images for: Identification of novel biomarkers involved in doxorubicin-induced acute and chronic cardiotoxicity, respectively, by integrated bioinformatics
Source: Front Cardiovasc Med. 2023 Jan 11;9:996809. doi: 10.3389/fcvm.2022.996809 (PMC9874088; doi:10.3389/fcvm.2022.996809)

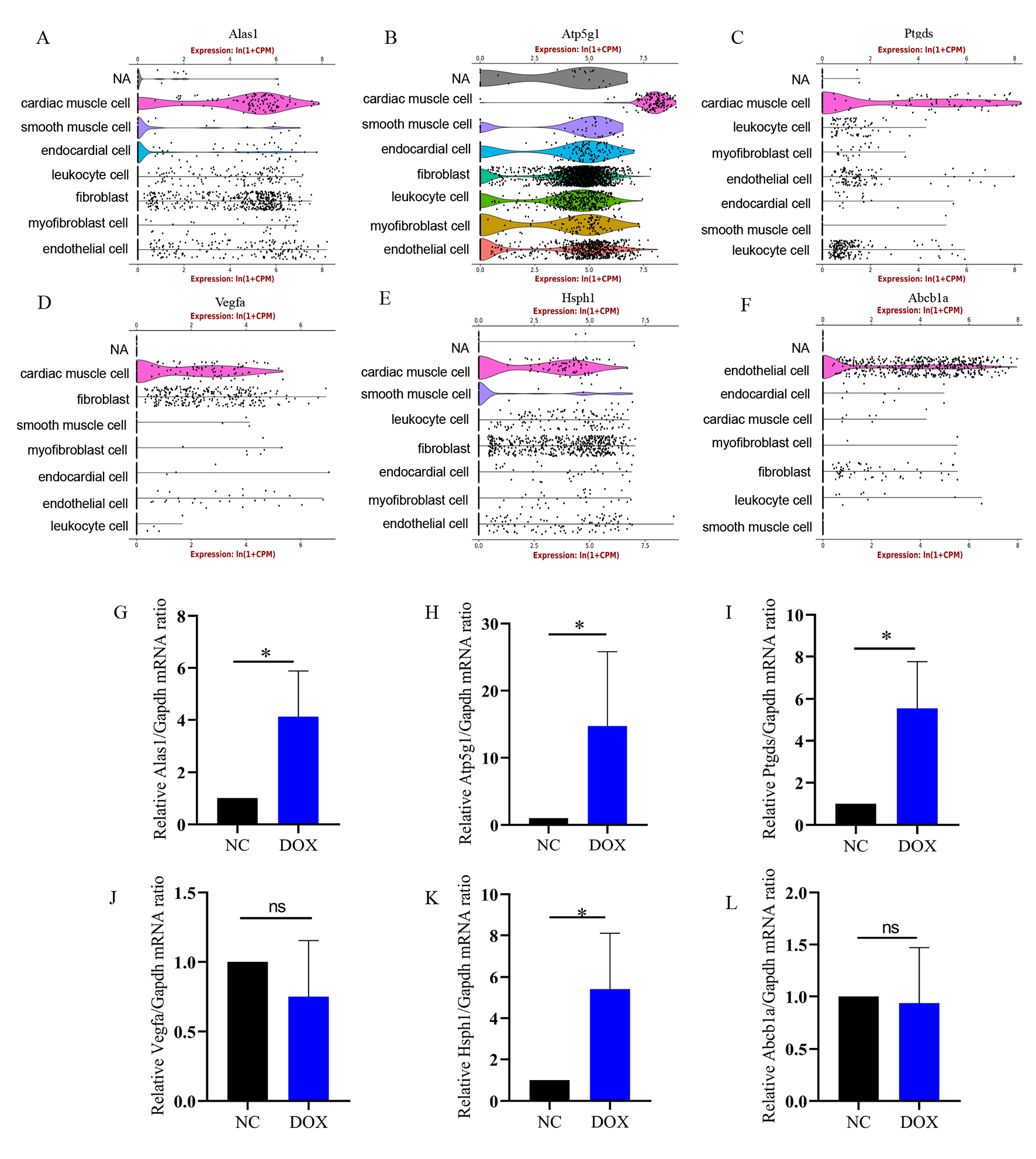

Supplement: Supplementary file 2 [file Image_1.TIF]

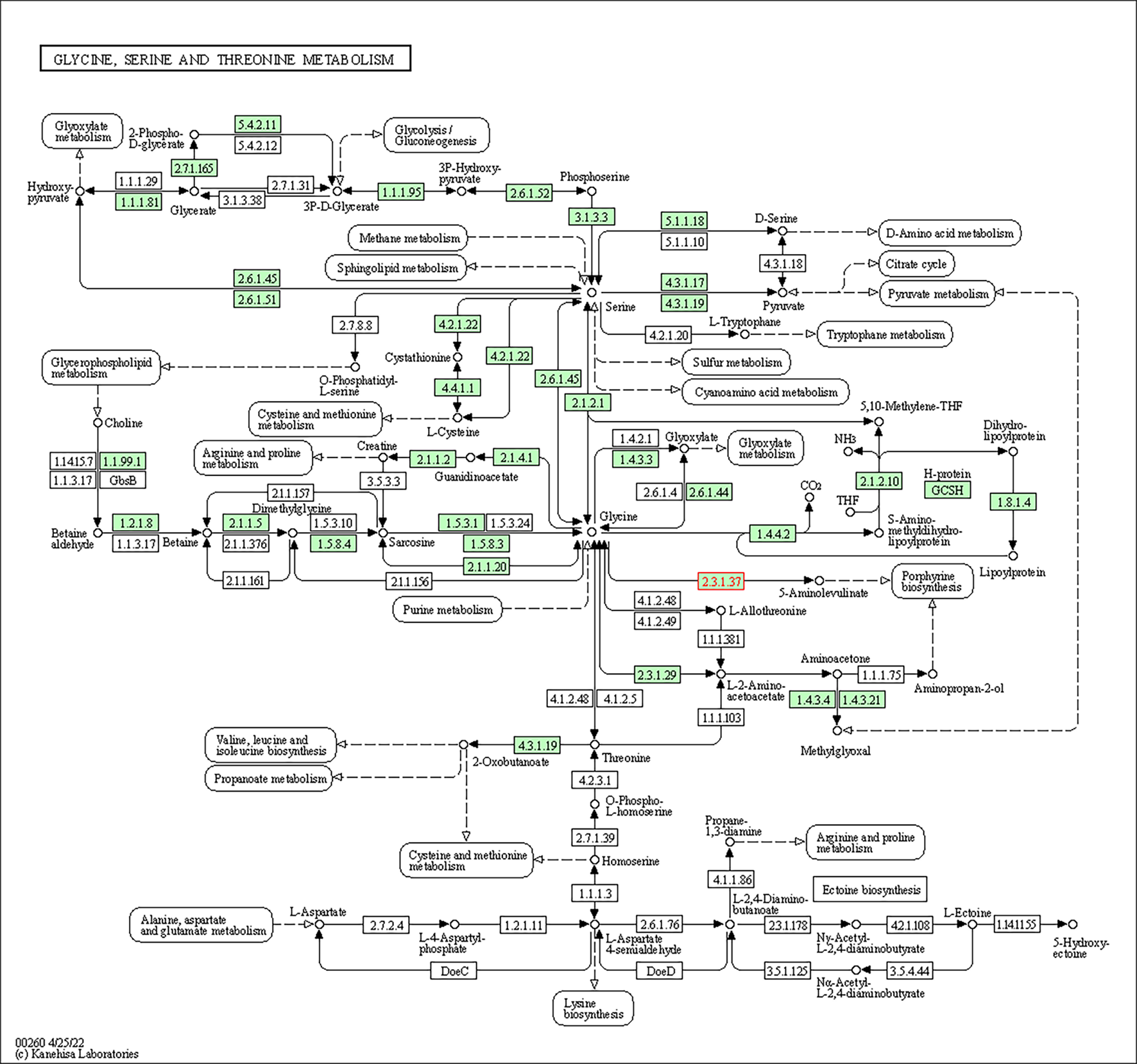

Supplement: Supplementary file 3 [file Image_2.TIF]

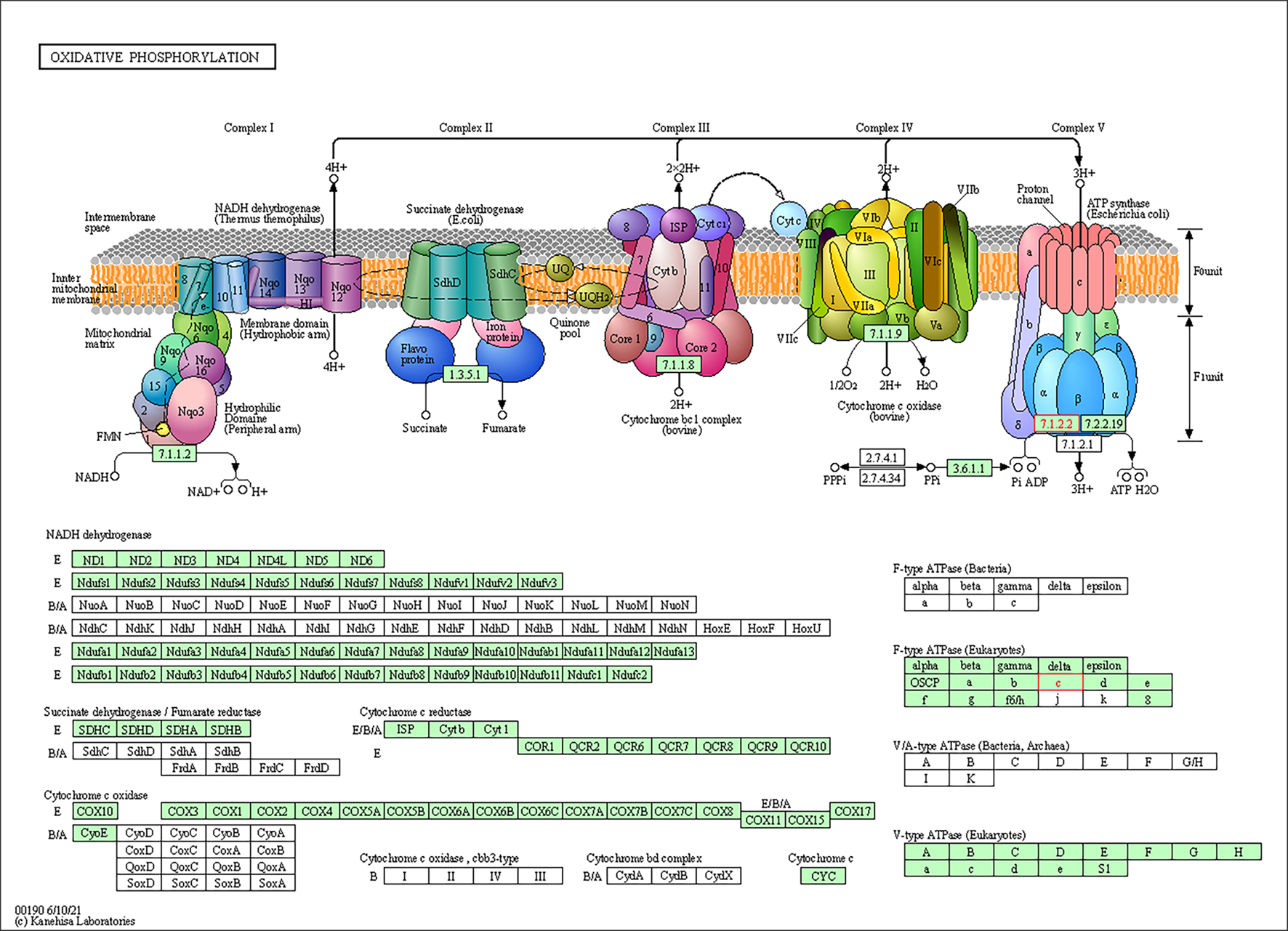

Supplement: Supplementary file 4 [file Image_3.TIF]

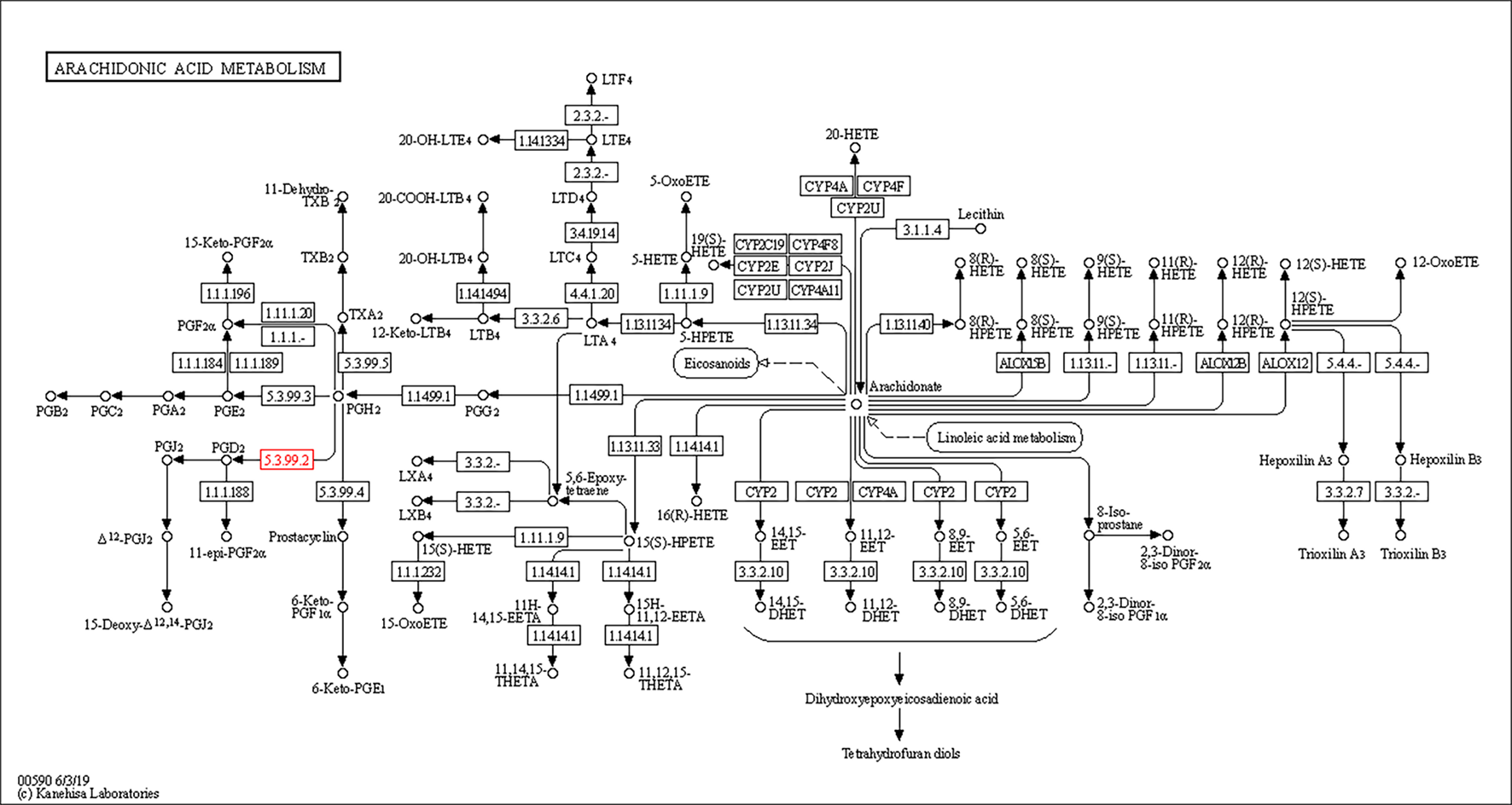

Supplement: Supplementary file 5 [file Image_4.TIF]

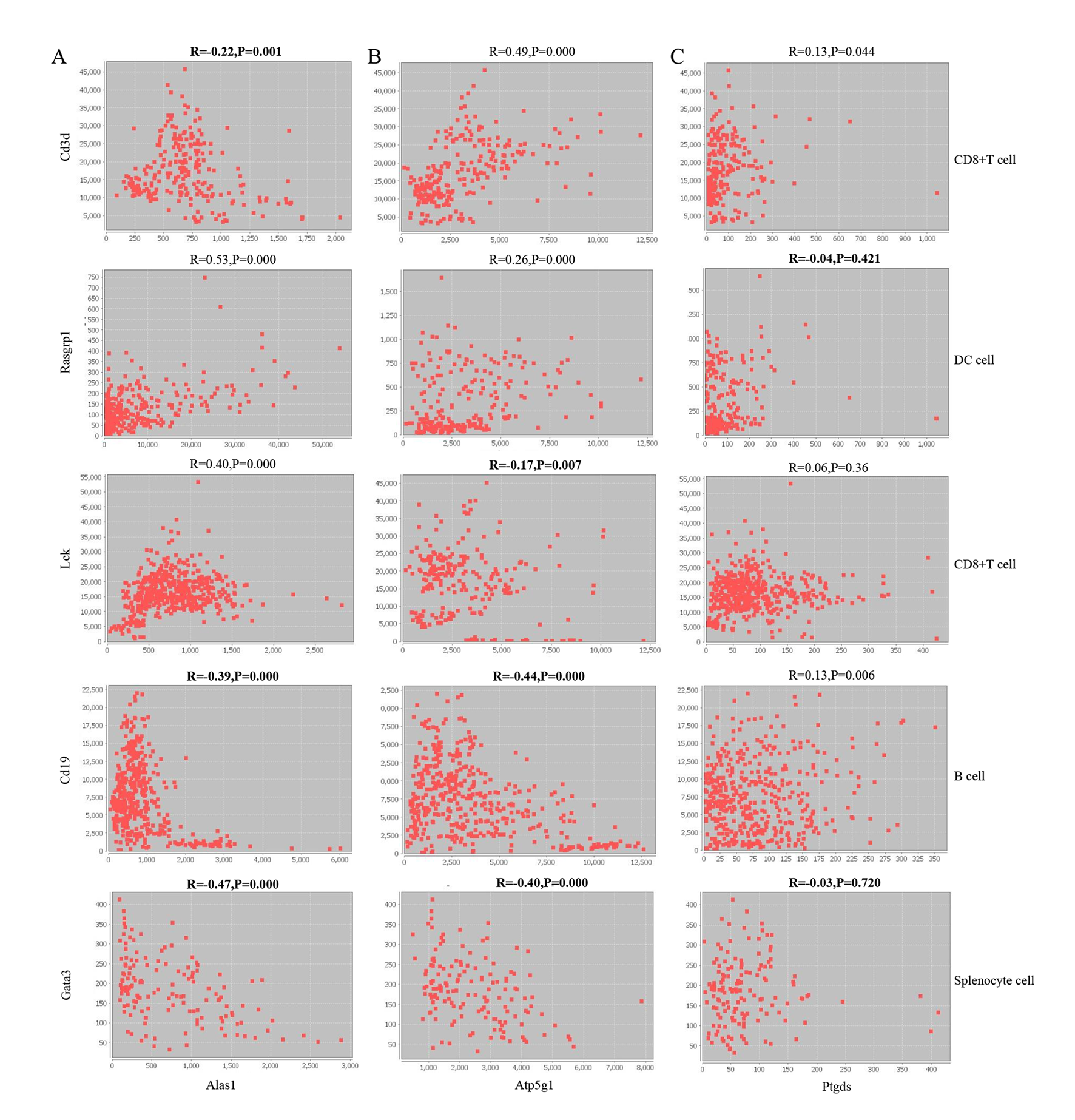

Supplement: Supplementary file 6 [file Image_5.TIF]

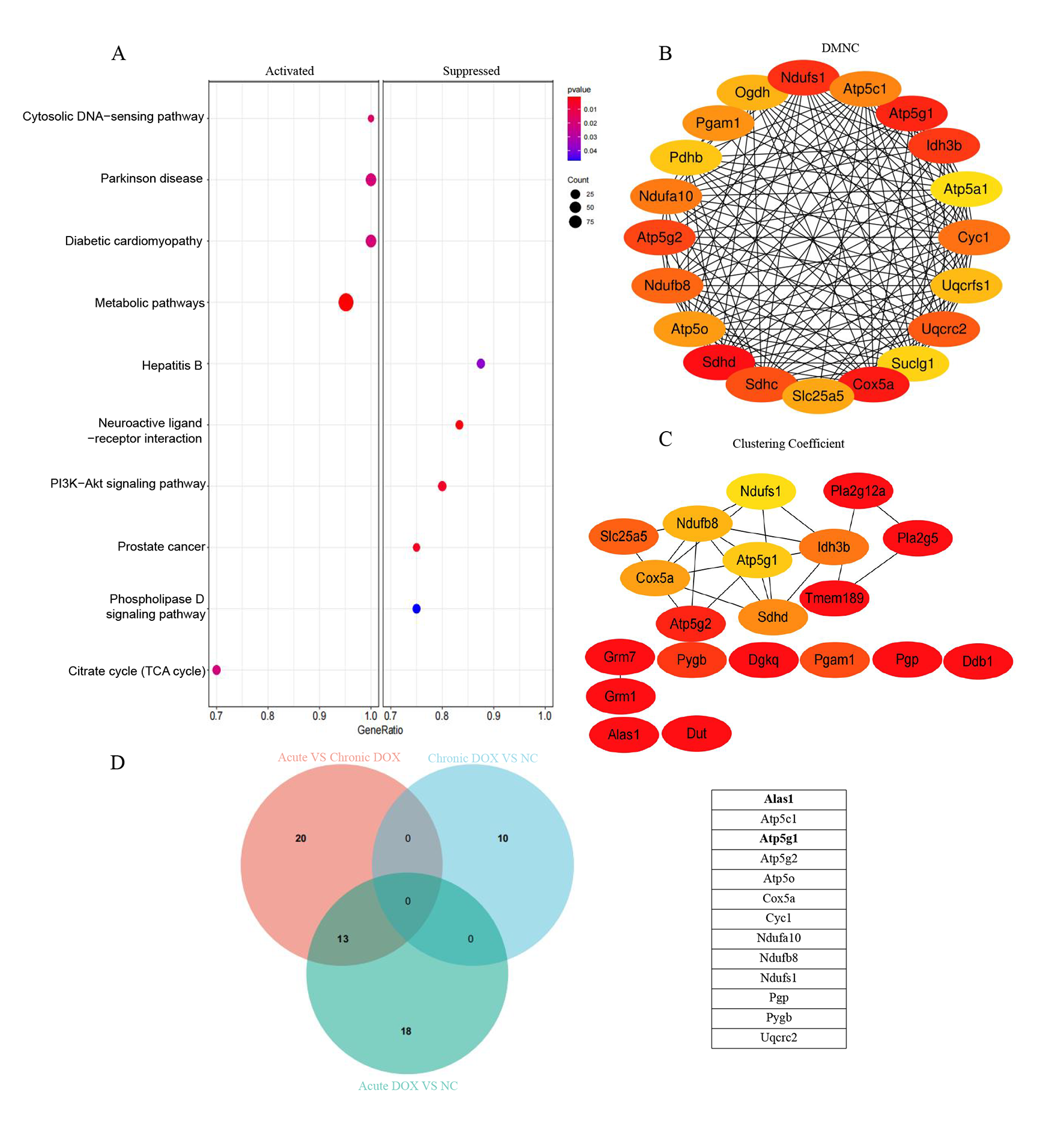

Supplement: Supplementary file 7 [file Image_6.TIF]
